# Letter from the Editor-in-Chief

**DOI:** 10.19102/icrm.2017.080505

**Published:** 2017-05-15

**Authors:** Moussa Mansour


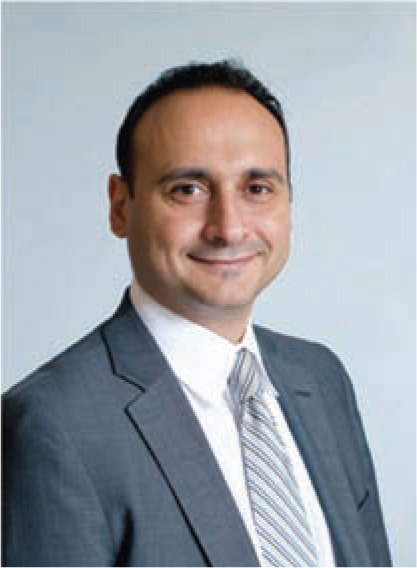


Dear Readers,

This issue of the *Journal* contains an important article by Drs. Epstein and Maytin titled “Strategies for Transvenous Lead Extraction Procedures.” In it, the authors discuss the indications for transvenous lead extraction (TLE), and include a summary of the 2009 HRS Expert Consensus Statement on Transvenous Lead Extraction. They also cover different technologies for TLE, including recently developed rescue tools, such as the SVC occlusion balloon. More importantly, the authors share helpful specific procedural instructions, illustrated in clear diagrams, and provide recommendations for the content of a “lead extraction cart.”

Over the past few years, TLE has become an integral part of the specialty of cardiac electrophysiology, and the number of extractions performed has increased significantly. This increase is driven by several factors, which include: (1) the fact that older and sicker patients are undergoing device implantation, leading to higher rates of infections and subsequent extractions; (2) the increased incidence of lead malfunction; and (3) the continuous, albeit slow, increase in the number of devices being implanted. As a result, it has become critically important for every electrophysiologist caring for patients with pacemakers and ICDs to be adequately informed with respect to all aspects of lead extraction, including indications for the procedure and the complications associated with it, in order to make the correct referrals when faced with an instance of infection or lead malfunction.

Lead extraction can be associated with significant morbidity and mortality. Operator and center experience play an important role in reducing complications. A multidisciplinary approach is very helpful, and is a model used in our center and by others. The “team” typically includes an electrophysiologist, a standby cardiac surgeon, and an anesthesiologist with transesophageal echocardiography experience, who perform the extraction procedure in a hybrid operating room. In addition, the availability of new technologies and rescue tools can make for a significant difference in the success of the procedural outcome.

I hope that you enjoy reading the above-mentioned article, as well the other articles in this issue of *The Journal of Innovations in Cardiac Rhythm Management.*

Sincerely,


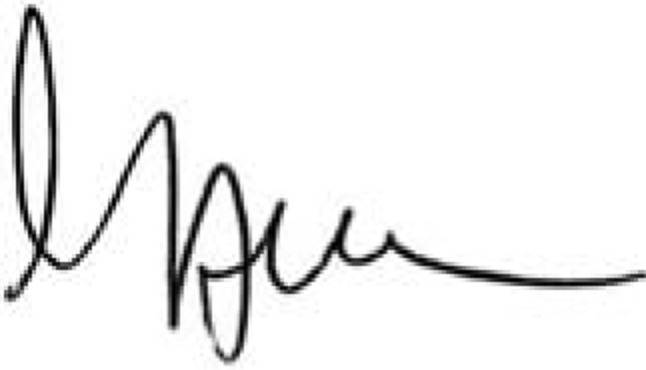


Moussa Mansour, MD, FHRS, FACC

Editor-in-Chief

The Journal of Innovations in Cardiac Rhythm Management

MMansour@InnovationsInCRM.com

Director, Cardiac Electrophysiology Laboratory

Director, Atrial Fibrillation Program

Massachusetts General Hospital

Boston, MA 02114

